# Supplementary material for: Interaction-free measurements by quantum Zeno stabilization of ultracold atoms
Source: Nat Commun. 2015 Apr 14;6:6811. doi: 10.1038/ncomms7811 (PMC4403339; doi:10.1038/ncomms7811)
Supplement: Supplementary Information — Supplementary Figures 1-3 and Supplementary Note 1 [file ncomms7811-s1.pdf]

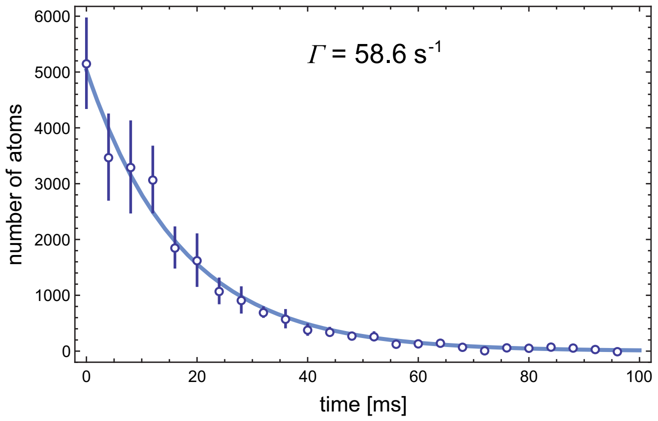

Supplementary Figure 1 | Calibration of the loss rate. Mean number of particles in the state $(1,-1)$ versus the exposure time. For this exemplary light intensity an exponential fit to the data (blue solid curve) yields a loss rate of $\Gamma = 58.6\,\mathrm{s}^{-1}$.

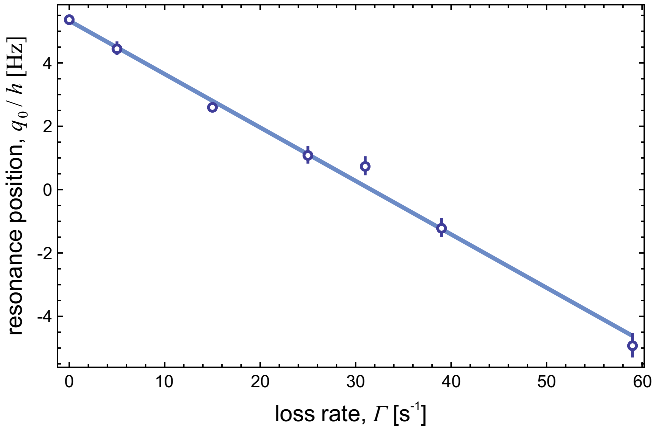

Supplementary Figure 2 | Resonance position versus loss rate. The position of the spin dynamics resonance is shifted depending on the loss rate Γ. The solid blue line is a linear fit to the data.

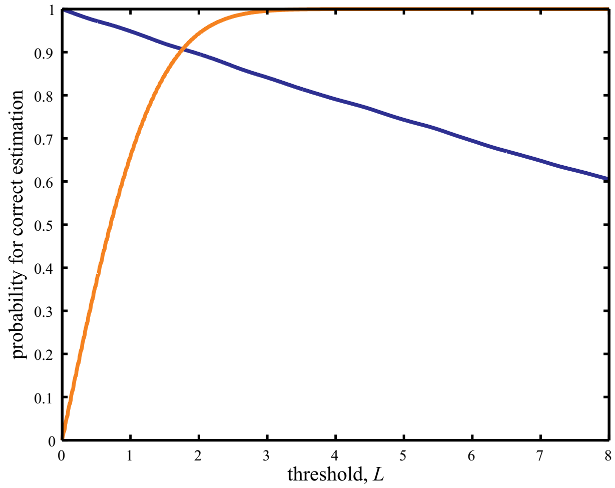

Supplementary Figure 3 | Discrimination probabilities as a function of the threshold L. In the "with object" ("without object") case, the orange (blue) line represents the probability that a single measurement result lies inside (outside) the range from $-L$ to L.

SUPPLEMENTARY NOTE 1: ELITZUR-VAIDMAN FIGURE OF MERIT

In this section, we derive a figure of merit for the demonstrated interaction-free measurements in analogy to the original Elitzur-Vaidman (EV) proposal [2]. In the EV proposal, the measurement is repeated until the photon either interacts with the object or exits the dark port D (see Fig.1a). The probability of an interaction-free measurement is thus given by

$$\begin{aligned}\eta &= P(\mathrm{D}) + P(\mathrm{B})P(\mathrm{D}) + P(\mathrm{B})^2 P(\mathrm{D}) + ... \\ &= P(\mathrm{D})\left[1 + P(\mathrm{B}) + P(\mathrm{B})^2 + ...\right] = \frac{P(\mathrm{D})}{1 - P(\mathrm{B})},\end{aligned}$$

where we summed the geometric series after an infinite number of trials. Taking into account that $P(\mathrm{D}) + P(\mathrm{B}) + P(\mathrm{int}) = 1$, we recover the familiar figure of merit

$$\eta = \frac{P(\mathrm{D})}{P(\mathrm{D}) + P(\mathrm{i}nt)}.$$

We generalize the figure of merit for the experimentally relevant case that an object can only be detected with a finite confidence. In our case, it is necessary to define a threshold L to discriminate between the presence and the absence of the object after a single measurement result. As in the original proposal, these measurement results are only evaluated if no interaction with the object took place.

Supplementary Figure 3 shows the probability that a single measurement result lies inside the range from $-L$ to L for the case with object (orange line). The probability increases monotonously from zero if $L = 0$ to almost 100% for $L > 4$. The blue line represents the probability that a single measurement result lies outside the range from $-L$ to L for the case without object. It decreases slowly and almost linearly from 100% at $L = 0$. At $L = 1.7$, the two probabilities become equal: This threshold is optimal in the sense that both cases are treated symmetrically. For this threshold, we achieve a confidence of 90% for both the presence and the absence of the object.

The EV proposal considers three different outcomes for the case with object: (i) interaction with the object, (ii) interaction-free measurement of the object, and (iii) inconclusive outcome. These three results correspond to the following three outcomes in our experiments: (i) interaction with the object with a 33% probability, (ii) a measurement result within -1.7 to 1.7, 60%, and (iii) a measurement result outside, 7%. The EV proposal requires a repetition of the measurement in case (iii). The figure of merit η is then calculated as the probability to detect an existing object without interaction after a conclusive series of measurements. For our confidence of 90%, we obtain a corresponding figure of merit $\eta = 65(2)\%$. Of course, this figure of merit could be further improved at the expense of a lower confidence for the "without object" case.
